# Notes and News

**Published:** 1892-05-07

**Authors:** 


### Notes and News.

COLLECTED AND COMMUNICATED.

We desire to direct attention to the account of four months spent in a provincial hospital, which was commenced in the "Nursing Mirror" last week. These sketches are not only interesting but practical, and we commend them to the consideration of all who care to gain a good insight into the daily routine of our large hospitals.

Amongst the many hundreds of people who are engaged in the management of the hospitals and asylums of this country there must be some with literary gifts and sound knowledge who might usefully aid the cause by writing from time to time upon points which arise in the administration of these institutions. Hospital administration is a wide subject, and we should gladly welcome any new hands who may tender their services, asking merely that any such offers shall be made only by those who are technically qualified and who have knowledge and experience to deal with the questions about which they may be moved to write.

Most hospital workers know Canon Erskine Clarke, of Battersea, by name, at any rate. No one has done better service for the sick and suffering, especially of the lower-middle class, than he has. Some years ago he established the Bolingbroke Pay Hospital, within an easy walk of Clapham Junction Station, advancing the necessary funds for the purpose, and he has ever since pluckily maintained it. Bolingbroke House provides for all cases where the means of the patients are small, and where also feelings of manly independence predominate. We could wish that those who take an interest in hospitals, and who have money at their disposal, could be induced to communicate with Canon Clarke and to visit Bolingbroke House. It is at the present time in need of some £3,000, and if that money is provided, not only will Canon Clarke be relieved from a heavy personal responsibility, which ought not to attach to him, but the prosperity and permanence of this much-wanted institution will be secured. We can state from personal knowledge that the nursing, medical care, and general arrangements of Bolingbroke House are excellent; and the fact that Mr. Thomas Bryant, the President of the Royal College of Surgeons, is the consulting surgeon, and takes an active interest in the progress of the institution, is in itself a guarantee of excellence.

An account of the proceedings at the Court of Governors of the Radcliffe Infirmary, Oxford, will be found in another column. We propose to deal with the points raised in our leading column next week. Meanwhile, it will be noticed that the proposal to institute an independent enquiry on the lines proposed by Sir Henry Acland, which, we hope will be followed, found favour with several speakers, although Mr. Fletcher's resolution was rejected.

The City of London Truss Society will hold its eighty-fifth annual festival on Tuesday next, the 10th inst., at the Albion Tavern. The Right Hon. the Lord Mayor will preside, and will be supported by the Sheriffs and a distinguished company of stewards. This charity is one of the oldest and most vigorous of its kind. Nearly 10,000 instruments were supplied gratuitously during 1891, some of which were very costly. Close upon half-a-million of poor patients have been relieved by the society.

Our attention has been called to a spirit of carping criticism which prevails amongst a certain class of people as an attitude of mind which they invariably adopt towards those who give much personal service to their fellows. If such small fry would remember that what the charities need nowadays is more cash and less criticism, they would strive to reach sounder ground, and, abandoning their carpings, would, let us hope, learn to give a shove to the coach, instead of impeding the progress of the horses who have at present all the work to do.

The workingmen of London have declared through their representatives that they are determined to get a *quid pro quo* for the money which they give to the hospitals. Unfortunately, there is amongst them a feeling that the hospital is the place for the workingman, and that he should invariably use it when members of his family or he himself are ill, because he believes the medical aid which he can afford to pay for is not of the best. It may be a remarkable fact, but we believe it to be true, notwithstanding, that the establishment of special hospitals of a certain class has had much to do with this feeling. Some representative working men hold the view that there ought to be a specialist for every disease to which flesh is heir, and that the man who does not seek out and fit the right specialist to his complaint is, to say the least of it, a noodle. On the other hand, it is the opinion—an opinion, by the way, which is based upon precise knowledge and long experience—of the leaders of the medical profession, that all sensible people do well to entrust themselves when ill to the care of a reputable general practitioner. This is so, because the general practitioner makes it his business out of his wide experience to understand how to apply everything which will tend to make his patients more comfortable, and so to promote their rapid recovery. It is time that the medical profession aroused itself, not only in its own interests, but in the interests of the people, and endeavoured to inculcate a more intelligent feeling amongst the artisan classes in regard to the best methods of treatment. Great as are the advantages of hospital treat- ment, in many instances we believe that the American sentiment, which holds that every man when ill is best at home in his own bed, is a sound one, and that it will be acted upon wherever possible by the more intelligent members of the community.

